# Supplementary material for: Glycoproteomics in Cerebrospinal Fluid Reveals Brain-Specific Glycosylation Changes
Source: Int J Mol Sci. 2023 Jan 18;24(3):1937. doi: 10.3390/ijms24031937 (PMC9916115; doi:10.3390/ijms24031937)
Supplement: Supplementary file 1 [file ijms-24-01937-s001.zip › ijms-2150913-supplementary-resubmitted-1-pdf/supplement_S3.pdf]

Supplementary information

Glycoproteomics in cerebrospinal fluid reveals brain-specific glycosylation changes

Melissa Baerenfaenger[1, 2]; Merel A Post[1]; Pieter Langenhorst[3]; Karin Huijben[3]; Fokje Zijlstra[3]; Joannes FM Jacobs[3]; Marcel M Verbeek[1, 3]; Hans JCT Wessels[3]; Dirk J Lefeber[1, 3,*]

[1] Department of Neurology, Donders Institute for Brain, Cognition, and Behavior, Radboud University Medical Center, Nijmegen, Netherlands
[2] Division of BioAnalytical Chemistry, AIMMS Amsterdam Institute of Molecular and Life Sciences, Vrije Universiteit Amsterdam, Amsterdam, Netherlands
[3] Translational Metabolic Laboratory, Department of Laboratory Medicine, Radboud University Medical Center, Nijmegen, Netherlands
* Correspondence: Drik.Lefeber@radboudumc.nl

This document contains supplementary Table S3.

Table S3. Patient information

CDG /Diagnose	Sex	Birth	Transferrin isoelectric focussing results	Clinical symptoms	Date of sampling
Alcohol abuse	M	24.06.1951	No information	No clinical symptoms described except for high fever.	18.07.1997
ALG1	M	03.12.1996	No information	No information	No info
ALG6	M	26.09.1995	Strongly deviating pattern with increase in asialo and disialo TF (19.08.2009)	Mental disorder, motoric retardation, seizures, epilepsy, abnormal physical appearance, lethargy, hypotony, feeding problems, recurrent infections.	15.12.1997
ATP6VoA2	F	25.09.1995	No information	Mental retardation, motoric retardation, abnormal fat distribution, facial myoclonus, *Curtis laxa*.	23.06.1998
DPM1	V	01.04.1998	No information	Serious neurological problems.	25.08.1999
DPM3	F	06.08.1981	deviating pattern with increase in disialo TF (19.03.2018)	Mental retardation, developmental delay, myopathy, cardiomyopathy, stroke-like episodes.	01.10.2001
MPI	F	05.12.1999	Deviating pattern with increase in disialo TF before treatment (01.03.2005). Dietary intervention with mannose was started in 2005. Subsequently, TF pattern normalized (10.01.2012).	Behavioural abnormalities, epilepsy/ epileptiform activity in EEG, dementia, thrombosis.	02.07.2008
NANS	M	21.12.2012	Normal TF pattern	Increased ManNAc levels in plasma, Skeletal dysplasia, delayed motor skills, Generalized hypotonia, Ventriculomegaly; Abnormality in periventricular white matter; Cerebral atrophy; Persistent vacuum vergae; Abnormal basal nuclei; Hypoplasia of the corpus callosum and splenium, *aplasia rostrum*; Asymmetry of cerebellum.	2014
no diagnose I	F	29.09.1995	No information	Suspected CDG	30.01.1998

no diagnose II	M	14.02.1995	Normal TF pattern (05.03.2009)	Unknown diagnosis, mental disorder, spastic-ataxic syndrome, ataxia, white matter abnormalities, cerebellar hypoplasia. Patient has a brother with the same clinical phenotype.	23.10.1997
PMM2_a	M	13.11.1997	No information	No information	16.04.1998
PMM2_b	M	24.02.1995	Slightly deviating TF pattern with increase in disialo TF (15.02.2006)	Motoric retardation, cerebellar hypoplasia, developmental delay, hypotony, microcephaly.	28.01.1998
SLC35A1	F	27.10.1977	Deviating TF pattern (06.03.1997)	Epilepsy, Consanguinity, imbecility, unexplained psychomotor disability. Patient died 15.12.1998.	23.01.1998
SRD5A3	M	16.10.1991	deviating pattern (06.03.1997)	Cerebral ataxia, coordination problems, developmental delay, muscular hypotonia.	17.02.1998
